# What Place Is There for Long-Acting Antibiotics in the Management of Gram-Positive Infections? A Qualitative Cross-Sectional Study

**DOI:** 10.3390/antibiotics13070644

**Published:** 2024-07-12

**Authors:** Aurélien Dinh, Guillaume Béraud, Johan Courjon, Yann Le Goff, Nicolas Kader Ettahar, Matthieu Grégoire, Eric Senneville

**Affiliations:** 1 Service des Maladies Infectieuses et Tropicales, Hôpital Raymond-Poincaré, 92380 Garches, France; 2 Service de Maladies Infectieuses, CHU d’Orléans, 45100 Orléans la Source, France; guillaume.beraud@chu-orleans.fr; 3 Service des Maladies Infectieuses et Tropicales, Université Côte d’Azur, CHU Nice, 06200 Nice, France; courjon.j@chu-nice.fr; 4 Public Health Expertise, 75004 Paris, France; yann.legoff@ph-expertise.com; 5 Unité de Maladies Infectieuses et Tropicales, CH de Valenciennes, 59300 Valenciennes, France; ettahar-n@ch-valenciennes.fr; 6 Cibles et Médicaments des Infections et de L’immunité, CHU Nantes, Nantes Université, 44000 Nantes, France; matthieu.gregoire@chu-nantes.fr; 7 Service Universitaire des Maladies Infectieuses et du Voyageur, Centre Hospitalier Tourcoing, 59208 Tourcoing, France; esenneville@ch-tourcoing.fr

**Keywords:** oritavancin, long half-life lipoglycopeptides, long-acting antibiotics, acute bacterial infections, Gram-positive, hospital discharge

## Abstract

Objectives. To identify the current practices with long half-life lipoglycopeptides (LGPs) and potential use/position of oritavancin. Results. Despite their indication being limited to skin and soft tissue infections (SSTIs), long half-life lipoglycopeptides are mainly used off-label to treat bone and joint infections (BJIs) and infective endocarditis. Oritavancin and dalbavancin are both semisynthetic lipoglycopeptide antibiotics with activity against Gram-positive organisms. The game-changing property of these two antibiotics is their one-time dosing. Due to its shorter half-life, oritavancin might have an advantage over dalbavancin for a treatment duration of less than 2 weeks, as it could be used both in prolonged treatments of complicated patients in BJIs or administered as a single-dose treatment for Gram-positive cocci infections usually treated by a 5- to 10-day antibiotic course. These infections include urinary tract infections, bacteremias, catheter-related infections, etc. In addition to the possibility of being used as an end-of-treatment injection, oritavancin could be used as an empiric therapy treatment in the postoperative period in the context of device-associated especially prosthetic joint infections to allow for the early discharge of the patient. Methods. A qualitative survey was conducted in March 2022 including sixteen infectiologists, one internist, five hospital pharmacists, and one pharmacologist. Conclusion. Long half-life lipoglycopeptides contribute to changing the paradigm in the management of acute bacterial infections, as infectiologists now consider a range of indications and patient profiles for one single drug. Oritavancin strengthens the therapeutic arsenal in numerous infections from BJIs to urinary tract infections and could help to manage specific clinical situations, on top of providing potential benefits for the hospital’s budget.

## 1. Introduction

Over the past few years, there has been growing interest in long half-life antibiotics, and among antibiotics, those active on Gram-positive bacteria have attracted interest, including methicillin-resistant *Staphylococci*, especially to treat bone and joint infections (BJIs), endocarditis, and skin and soft tissue infections (SSTIs).

Oritavancin is a new long-acting semisynthetic lipoglycopeptide that can be administered as a single dose to treat SSTIs because of its extensive terminal half-life (around 10 days) [1,2,3]. Oritavancin activity on Gram-positive bacteria is supported by three mechanisms of action: (i) inhibition of the transglycosylation or polymerization step of cell wall biosynthesis by binding to the stem peptide of peptidoglycan precursors; (ii) inhibition of the transpeptidation or crosslinking step of cell wall biosynthesis by binding to the peptide bridging segments of the cell wall; and (iii) disruption of bacterial membrane integrity, leading to depolarization, permeabilization, and rapid cell death [4]. It also displays a rapid concentration-dependent bacterial killing [5]. In a recent study, it has been demonstrated that oritavancin exerts rapid bactericidal activity against methicillin-resistant *Staphylococcus aureus* (MRSA) isolates that are maintained in stationary-phase state in vitro, whereas dalbavancin and vancomycin do not [6], notably due to their wide tissue distribution with a volume of approximately 1 L/kg and accumulation in macrophages, reaching intracellular concentrations 200 times higher than extracellular concentrations after 24 h of incubation in vitro [7,8]. These results provide some reassurance that oritavancin accumulation does not prevent phagocyte killing and suggest that this antibiotic may help eradicate staphylococci within cells [9].

In a 2-year, multicenter, retrospective, descriptive study of 134 patients treated for acute osteomyelitis, 4 to 5 doses of oritavancin (1200 mg followed by 800 mg once per week for 3 or 4 weeks) showed positive clinical outcomes—high rate of clinical success (88.1%) and low rate of adverse events (3.7%) [10]. Oritavancin safety and tolerance was also highlighted in other real-life studies [11,12].

Oritavancin has been approved since 2015 in the European Union (2014 in the USA) and has recently become available in France, so we conducted a qualitative survey consisting of 1 h interviews with a selection of key French experts to identify oritavancin’s place and to anticipate its best use.

## 2. Results

### 2.1. Respondents and Facilities

**Figure 1:**
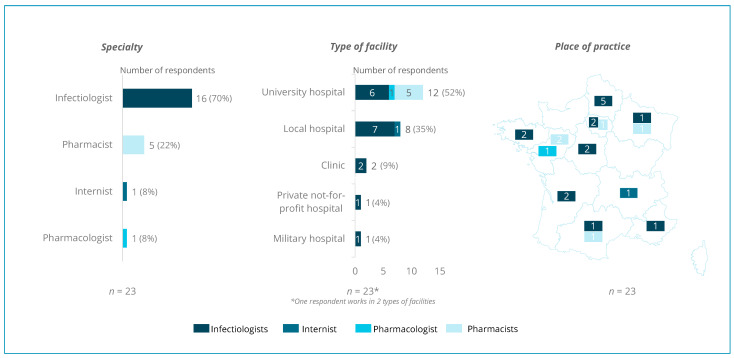
Sample description.

A panel of sixty infectious disease specialists was contacted by email, and twenty-three (38%) agreed to be interviewed. Among them, 21 were men and 2 were women. Those healthcare professionals from four medical specialties (sixteen IDSs, one internist, five hospital pharmacists, and one pharmacologist) that practice in different types of French facilities (52% university hospitals, 35% local public hospitals, 9% clinics, one private non-profit hospital, and one military hospital) agreed to be interviewed (Figure 1) and have different types of profiles: some of the investigated facilities of the panel are considered as the referral center in infectious disease in their geographic area, while others see a smaller number of patients.

A total of 91% of the survey respondents are members of the Anti-Infectives Committee (CAI/COMAI), 65% are members of the Medication and Sterile Medical Devices Committee (COMEDIMS), and 48% are members of the Institutional Medical Committee (CME). Finally, nine (39%) work in a referral center for bone and joint infections with a high degree of expertise in the field.

### 2.2. Protocols

Written treatment protocols or medical guidelines are not universally established for all types of indications across the investigated facilities. Among the respondents, 18 out of 23 (78%) reported having a written protocol for device-related bone and joint infections (BJIs) at their facility. Additionally, 17 respondents (74%) confirmed protocols for BJIs without orthopedic devices and for postoperative empirical antibiotic treatment (AB-prophylaxis). Protocols for skin and soft tissue infections (SSTIs) were reported by 16 respondents (70%). However, the presence of protocols is less consistent for the management of osteomyelitis (52%) and infective endocarditis (50%).

Anesthesiologists participate in the writing of antibioprophylaxis treatment protocols alongside IDSs; in other indications, IDSs often draft the protocols with pharmacists and microbiologists. All respondents include a description of the dosing regimens in the protocols, while 39% include patient profile descriptions.

The modification of the treatment protocols for Gram-positive bacterial infections is mostly driven by the evolution of data and the availability of new molecules (48% of respondents), the ecology of the facility/the evolution of bacterial resistance (43%), and the new guidelines (43%). It is often a multidisciplinary discussion within the anti-infectives committee or a dedicated meeting between experts that leads to the update of a treatment protocol. Specialists’ adherence to the written protocols is relatively high, with an estimated average of 86% in investigated centers: 50% adherence at the lowest and 100% at the highest level.

### 2.3. Place of Long Half-Life Lipoglycopeptides in the Therapeutic Strategy

**Figure 2:**
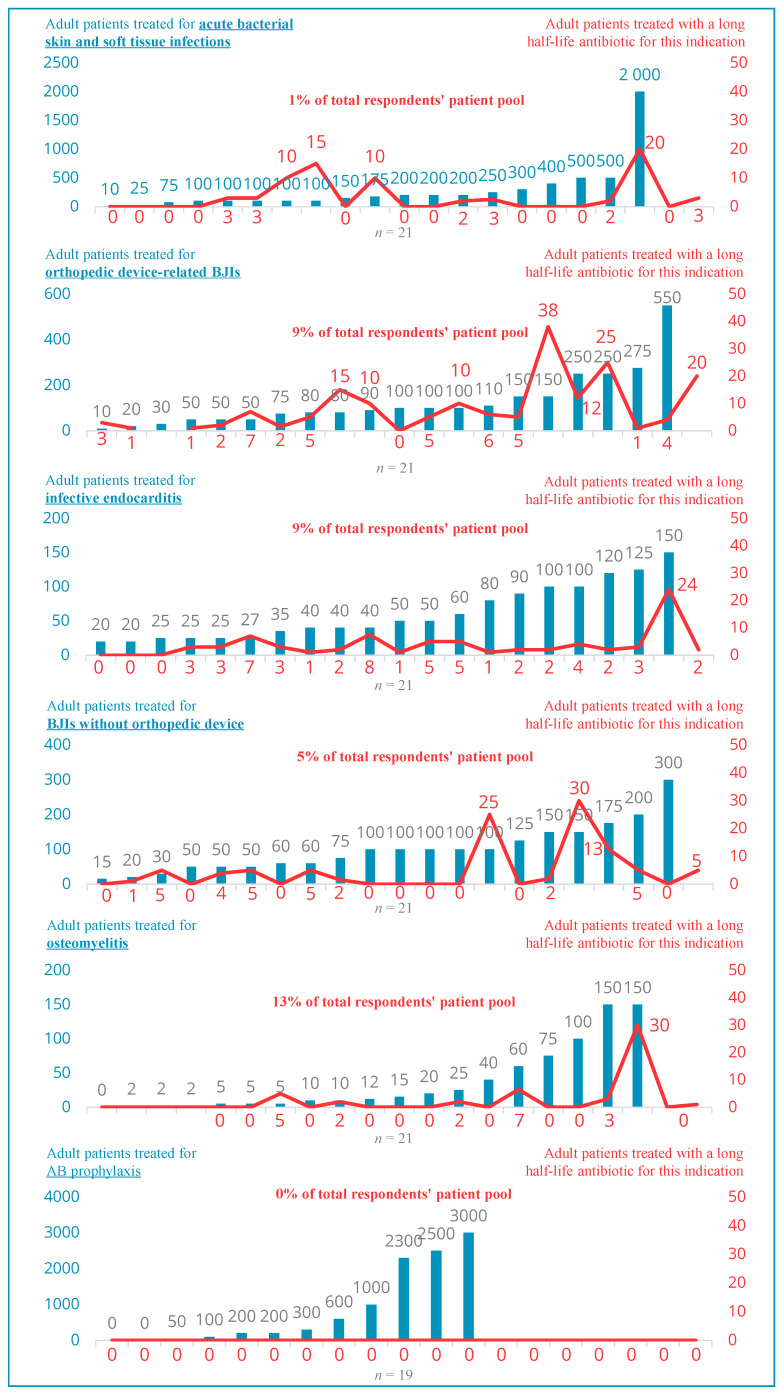
Indications (Supplementary Materials, File S1).

Heterogeneities between investigated healthcare facilities in the use of long-acting lipoglycopeptides (only dalbavancin) have been identified. Nine (9/22) institutions use these antibiotics in the authorized indication (i.e., ABSSSIs), but for a limited number of patients representing <1% of the total pool of adult patients (Figure 2).

Regarding off-label use (Figure 2), long-acting lipoglycopeptides are used in the treatment of the following:

– Orthopedic device-related BJIs (prosthesis, implant, osteosynthesis, etc.) in 18 facilities for a total of 9% of the respondents’ total pool of patients in this indication and up to 30% in one center.
– Infective endocarditis in 17 facilities and for a total of 9% of the respondents’ pool in this indication.
– Osteomyelitis in six facilities and for a total of 13% of the respondents’ pool in this indication.
– For other BJIs without implanted device in 11 facilities and for a total of 5% of the respondents’ pool in this indication.

In an opened question, 6/23 respondents reported using long-acting antibiotics in the treatment of vascular infections (for 13% of their cumulated pool of patients), and 5 reported using them for catheter-related infections (for 3% of their cumulated pool of patients). Finally, one expert mentioned the treatment of mediastinitis and in the context of circulatory assistance and one other in suppressive.

Nevertheless, all of the respondents (*n* = 19) declared not to use this type of antibiotic in AB-prophylaxis.

**Figure 3:**
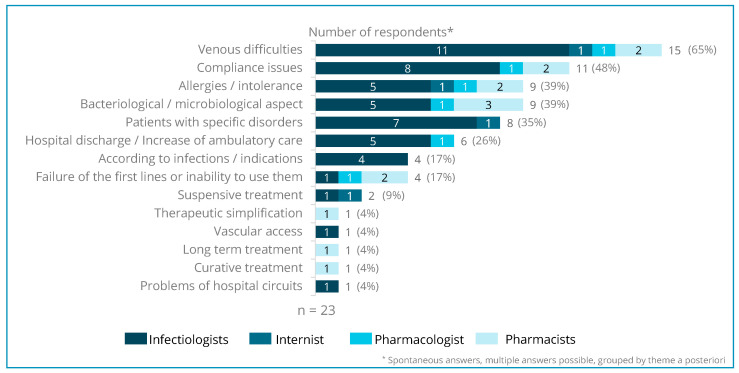
Patient profiles and/or clinical factors.

Physicians seem to mainly use long-acting lipoglycopeptide strategies for “complex” patients (57% respondents), as described in a French cohort [13]. Long-term strategies are often set up to treat infective endocarditis and BJIs with or without an orthopedic device. The term “complex patients” indicates problems of venous access, intolerance to first-line treatments, compliance issues, allergies, comorbidities, psychiatric issues, etc. (Figure 3).

Fifty-two percent (12/23) of respondents use this type of drug in other specific situations depending on microbiological results, as a suppressive treatment (long course), or as a salvage therapy (after several lines of treatment).

On the patient’s pathway, the first dose is mostly given during hospitalization, but it is common for the following injections to be performed during day-care hospitalization (for 17/22 (77%) of respondents), and sometimes in outpatients at follow-up and rehabilitation care (10/22 (45%)), or even hospitalization at home (10/22 (45%)).

The financing system for the drug also differs from one care facility to another. Indeed, financing is sometimes linked to the infectious disease department, sometimes to the prescribing service, and sometimes to the hospital. Some centers benefit from external funding sources.

IDSs must request authorization from the pharmacy in 2 out of 5 investigated centers to be able to use these antibiotics once they are listed in the hospital, but this is often very formal and simple to obtain the authorization with the description of the patient profile.

### 2.4. Place for Oritavancin: A New Long Half-Life Lipoglycopeptide

As of today, dalbavancin is the only long-acting lipoglycopeptide used in investigated facilities. It is listed and prescribed in all panel centers, except one private hospital, and stocks are usually available at the pharmacy. However, fourteen respondents out of the twenty-three (61%) believe it would be useful to have another long half-life antibiotic available in the facility. Indeed, eight of those fourteen respondents think the difference in half-life could lead to different patient profiles, four think that it is always good to have alternative antibiotics, three point out that different antibiotics could act on different types of bacteria/microorganisms, and two put forward the idea of the economic competition between the two drugs. Oritavancin could be a good alternative to dalbavancin for 52% of the interviewees who think there would be specific profiles for each drug. According to them, oritavancin would be favored for shorter treatments, in postoperative situations, or for specific indications such as Gram-positive cocci infections for which the recommended treatment duration is 5 to 10 days, or specific bacteria/microorganisms such as enterococci or staphylococci. The other 48% think that the products are interchangeable.

When there are several antibiotic alternatives to treat Gram-positive bacterial infections, physicians tend to choose based on the perceived efficacy (score of 5/5 on a 5-point scale with 0 = not taken into consideration to 5 = major), long-term tolerance (4.1), short-term tolerance (3.9), experts’ recommendations (3.9), economic criteria (3.7), and prescription habits (3.5). Conversely, interviewed experts do not seem to consider the services offered by pharmaceutical companies to healthcare professionals (2.2) or to patients (2.2), the authorized indications of the drug (2.2), the length of the injection (1.8), or the corresponding pharmaceutical company’s reputation (1.4).

### 2.5. Economic Considerations

Finally, 91% of the respondents report that antibiotic economic features are closely analyzed in their centers, in hospital pharmacies (91%), or even in the department (61%). However, the price indicator is not the same for all interviewed physicians; some consider the price per injection (39%), some take into account the price for the whole cure (30%), others consider the overall benefit of the drug (26%), meaning the total price of the treatment plus the reduction in the number of days of hospitalization, the increased quality of life, or prevention of venous infections, and 9% are more interested in treatment cost per day.

Thus, 83% of the respondents believe that a medico-economic demonstration of the benefit of long half-life antibiotics would help in their use; those who do not believe the medico-economic arguments would change their prescription behavior work in a clinic, a private hospital, and two in university hospitals.

## 3. Discussion

This survey of twenty-three healthcare professionals involved in the management of difficult-to-treat Gram-positive infections highlights the fact that the choice of an antibiotic is not only linked to a specific type of microorganism or a single indication but is multidimensional. In addition to the clinical situation, IDSs tend to take into consideration patient profile (fragility, capacity to be compliant, etc.), patient comfort (preference on the galenic form, early discharge from hospital), and hospital capacity (beds being released, medical time saving).

Thus, IDSs perceive the interest that long half-life antibiotics can have in their current practice. The benefit of long half-life lipoglycopeptides is particularly identified for specific patient profiles such as those for which a long hospitalization would be very difficult or unnecessary except for the administration of treatments. This reasoning can actually be extended to a larger number of patients knowing that the length of hospitalization is associated with complications and sometimes with a loss of autonomy and a lower quality of life [14].

However, the use of long-acting antibiotics very often comes up against problems of access within hospitals, even if the recent medico-economic studies which demonstrated an interest in these molecules have slightly changed people’s minds. Indeed, in a multicentric cohort based on the French registry of dalbavancin use in 2019 compared to the French national discharge summary database, Béraud et al. [15] show that dalbavancin is associated with early discharge due to its long half-life, especially in the case of early switching (administration within less than 11 days of hospitalization).

Physicians have also aroused interest in having molecules with different long half-lives. Thus, oritavancin could be used (i) by being administered as a one-shot treatment for Gram-positive *cocci* infections usually treated by a 5- to 10-day antibiotic course, (ii) as a postoperative empiric antibiotic treatment, and (iii) as a prolonged treatment for difficult-to-treat patients in various indications such as BJIs or endocarditis.

As stated by the scientific committee, the availability of two specific long-activity drugs instead of one limits the risk of shortage, and then a shorter long half-life would open up the field to numerous new indications, especially when oral treatments are not indicated because of the antimicrobial susceptibility profile of the microorganism(s) or the patient himself. For instance, oritavancin could be favored for infections requiring shorter treatments, such as urinary tract infections, catheter-related infections, in postoperative situations, or for specific indications such as Gram-positive cocci infections for which the recommended treatment duration is 5 to 10 days. The management of these infections would be optimized by a single-injection drug, especially since oral treatments can have low compliance rates, which is problematic for reasons of resistance selection and treatment failure [16].

Aside from its long half-life, oritavancin displays an interesting set of in vitro properties that are relevant in the context of chronic infections, including those involving orthopedic material. These in vitro properties could make a difference in the treatment of such infections. Indeed, the molecule demonstrated a fast concentration-dependent bactericidal activity on both planktonic and stationary phase bacteria [6], which distinguish it from other glyco- and lipoglycopeptides. Oritavancin also demonstrated a good efficacy on biofilm-associated SARM and methicillin-resistant *S. epidermidis* (MRSE) in vitro [17,18], and its spectrum of activity makes it an interesting option for specific microorganisms like multidrug-resistant enterococci, *Cutibacterium acnes*, or *Corynebacterium* spp.

In the context of empirical antibiotic treatment after revision procedure for suspected PJI, oritavancin may be suitable. According to the available data on microbial epidemiology, especially chronic prosthetic joint infection (PJI), among coagulase negative staphylococci (CNS), the rate of methicillin resistance can rise to 80% [19,20]. Furthermore, the availability of full microbiological data may require 5 to 11 days, or even more when considering *Cutibacterium acnes* [21,22]. Thus, one injection of oritavancin can appropriately cover this period even for an outpatient setting before reconsidering the diagnosis of PJI and the choice of a definitive antibiotic strategy for several weeks.

Oritavancin seems to be the proper choice in two specific clinical situations: (i) as a “one shot therapy” for patients who need less than 11 days of treatment and (ii) as a “finishing treatment” for patients who need an early discharge.

The consulted experts asked for additional PK-PD data to define more precisely the dosing regimen for reinjection when considering the long-term treatment of chronic infections, and they anticipate that this drug could be used by non-specialists once the administration regimens are better defined.

From the collective perspective and particularly in a context of the saturation of hospital wards in France and given the difficulties in ensuring the post-hospitalization management of patients, these molecules are perceived as innovations with a high degree of potential for organizational facilitation. Indeed, they can greatly streamline routes and limit traffic jams in medicine and infectious disease departments. Nevertheless, physicians need to appropriate these new therapeutic strategies and define therapeutic protocols for each indication so that management can be based on shared and validated recommendations.

One study already reports the use of oritavancin and dalbavancin in the real world. The main indications were skin and soft tissue infections, BJIs, and endocarditis, and the most common isolated pathogens were MSSA and MRSA. Hospital length of stay and health system costs were dramatically reduced [23]. A systematic review provides evidence that laLGPs are safe and efficacious for off-label use like osteoarticular, cardiovascular, intravascular-catheter-related, and other complicated infections [24].

### Limitations

Our study presents several limitations. It is a qualitative cross-sectional study focusing on a drug that currently lacks market access in France, and therefore does not reflect future clinical practice. Additionally, we only interviewed experts in the field, but not all of them were available for the study. Future research on the real-world use of long-acting lipoglycopeptides, particularly oritavancin, will be of significant interest.

## 4. Methods

A scientific committee of 6 infectious disease specialists (IDSs) was set up to support the qualitative survey. They oversaw the validation of the interview guide and the interpretation of the results. The experts involved are all practicing in different geographical areas across France.

The interview guide, written with the help of the scientific committee, included 40 questions and subquestions focusing on the following:

(a) The qualification of respondents;
(b) The protocols used;
(c) The place of long half-life lipoglycopeptides in the therapeutic strategy;
(d) The place for oritavancin;
(e) Economic considerations.

Key Opinion Leaders were selected using the Expertscape database—which ranks the top experts based on their number of publications—and an objective of selecting different types of specialties, a diversity of facilities, and a dispersion across the national territory.

Interviews were conducted by videoconference in March 2022, with infectiologists, hospital pharmacists, internists, and pharmacologists practicing in different types of French healthcare facilities. The interviews lasted approximately one hour.

The survey’s outcomes were discussed among the scientific committee members during a meeting in order to further validate the potential positioning of oritavancin in the current therapeutic armamentarium.

## 5. Conclusions

This survey of twenty-three healthcare expert professionals shows that, according to their personal perspectives, there is a possible place for oritavancin, the newest long half-life lipoglycopeptide, in the management of Gram-positive bacterial infections, especially for patients for whom a long hospitalization would not be appropriate or to accelerate hospital discharge.

Oritavancin will strengthen the therapeutic arsenal in numerous infections from BJIs to urinary tract infections or catheter-related infections. Its good efficacy on MRSA and MRSE biofilms can be an advantage in the context of device-associated prosthetic joint infections, and its microbiological spectrum makes it an interesting option for difficult-to-treat infections due to specific bacteria/microorganisms like resistant *enterococci*, *Cutibacterium acnes*, or *Corynebacterium* spp. infections. Thus, this new drug contributes to changing the paradigm in Gram-positive bacterial infections, with IDSs considering a range of indications and patient profiles. Real-world evidence from oritavancin use in France will reveal how the molecule actually fits into the current therapeutic armamentarium. To do so, a national registry of oritavancin use, describing efficacy and tolerability, will be of utmost interest [25].

## Data Availability

The data presented in this study are available on request from the corresponding author due to privacy, legal, or ethical reasons.

## Supplementary Materials

The following supporting information can be downloaded at: https://www.mdpi.com/article/10.3390/antibiotics13070644/s1, File S1: Indications.
