# Supplementary material for: What Place Is There for Long-Acting Antibiotics in the Management of Gram-Positive Infections? A Qualitative Cross-Sectional Study
Source: Antibiotics (Basel). 2024 Jul 12;13(7):644. doi: 10.3390/antibiotics13070644 (PMC11274069; doi:10.3390/antibiotics13070644)
Supplement: Supplementary file 1 [file antibiotics-13-00644-s001.zip › antibiotics-3043480-supplementary.pdf]

Supplementary File S1

I. Qualification of the respondent

1. Are you part of the COMEDIMS? ☐ Yes ☐ No
2. Are you part of the CME? ☐ Yes ☐ No
3. Are you part of the CAI / COMAI? ☐ Yes ☐ No

II. Protocols

4. In your hospital department, are there treatment protocols for:

• Acute bacterial skin or soft tissue infections? ☐ Yes ☐ No

• Bone and joint infections without implanted material? ☐ Yes ☐ No

• Bone and joint infections with implanted material (prosthesis, plate, etc.)? ☐ Yes ☐ No

• Osteomyelitis? ☐ Yes ☐ No

• Infective endocarditis? ☐ Yes ☐ No

• Prevention of postoperative infections? ☐ Yes ☐ No

If No, go to question 3.

If Yes,

4.1. Which actors are involved in the drafting of these protocols, in addition to infectious disease specialists? How?

4.2. What level of detail do these protocols contain?

• Descriptions of patient profiles: ☐ Yes ☐ No

• Dosages: ☐ Yes ☐ No

• Mention of the antibiotic by:

☐ Therapeutic class ☐ Brand name ☐ International Nonproprietary Name (INN)

If INN / brand name:

4.2.1. What factors allow these protocols to be modified?

4.2.2. What are the modalities for changing these protocols?

4.3. What is the adherence rate of specialists to this protocol...

• Within the center: ....%

• At the CRIOAC: ....%

III. Place of long half-life lipoglycopeptides in the therapeutic strategy

5. What is the pool of patients each year in the hospital department for these different indications? And how many of them are treated with long half-life antibiotics?

Number of patients treated each year	In the hospital department	With long half-life antibiotics
Acute bacterial skin or soft tissue infections		
Bone and joint infections without implanted material		
Bone and joint infections with implanted material (prosthesis, plate, etc.)		
Osteomyelitis		
Infective endocarditis		
Prevention of postoperative infections		
Other (specify)		

6. What therapeutic strategies do you consider appropriate for long half-life glycopeptide antibiotics in these indications? (Multiple choices possible)

- ☐ Long-term strategies for "complicated" patients
  - ☐ All indications
  - ☐ Acute bacterial skin or soft tissue infections
  - ☐ Bone and joint infections without implanted material
  - ☐ Bone and joint infections with implanted material (prosthesis, plate, etc.)
  - ☐ Osteomyelitis
  - ☐ Infective endocarditis
  - ☐ Prevention of postoperative infections
  - ☐ Other (specify): ....
- ☐ Postoperative prophylaxis strategies
  - ☐ All indications
  - ☐ Acute bacterial skin or soft tissue infections
  - ☐ Bone and joint infections without implanted material
  - ☐ Bone and joint infections with implanted material (prosthesis, plate, etc.)
  - ☐ Osteomyelitis
  - ☐ Infective endocarditis
  - ☐ Prevention of postoperative infections
  - ☐ Other (specify): ....
- ☐ None
- ☐ Other (specify)

7. What patient profiles and/or clinical factors lead you to prescribe a long half-life antibiotic instead of a "standard" IV antibiotic or an oral antibiotic for the treatment of gram-positive bacterial infections in the indications we have mentioned?

#1	
#2	
#3	
#4	
#5	

7.1. Specify if different profiles by indication

8. Do you currently use dalbavancin? ☐ Yes ☐ No

If Yes:

8.1. Is this treatment listed in the pharmacy? ☐ Yes ☐ No

8.2. Is there stock available in the pharmacy? ☐ Yes ☐ No

If No,

8.3. Why?

IV. Place of oritavancin in the therapeutic strategy

9. Oritavancin is a new antibiotic of the glycopeptide class, for systemic use, indicated for ABSSSI in adults, targeting Gram-positive bacteria (including MRSA). Are you familiar with this medication? ☐ Yes ☐ No

10. As of now, what is the likelihood that oritavancin:

• Will be listed in the pharmacy instead of dalbavancin in the hospital: ......%

• Will be listed in addition to dalbavancin in the hospital: ......%

• Will not be listed in the hospital: ......%

∑=100%

11. Do you think it would be beneficial for patients to have two long half-life antibiotics indicated for acute bacterial infections available in the establishment? ☐ Yes ☐ No

11.1.Why?

12. Do you need to request authorization from the pharmacy to use this type of medication once they are listed in the hospital? ☐ Yes ☐ No

If Yes:

12.1. What are the criteria for their use (type of patients, usage cap, etc.)?

13. Would a medico-economic demonstration help you in your choice to use long half-life antibiotics? ☐ Yes ☐ No

14. What are the patient profiles corresponding to each product (type of infection, socioeconomic criteria, treatment line, pathogen, venous route, etc.)?

Dalbavancin	
Oritavancin	

15. How do you evaluate the product characteristics of these two medications for the mentioned patient profiles, on a scale from 0 (very negative opinion) to 10 (very positive opinion) in terms of...

	Dalbavancin	Oritavancin
Efficacy	/ 10	/ 10
Tolerance	/ 10	/ 10
Ease of use	/ 10	/ 10
Overall score	/ 10	/ 10

15.1. For what reasons?

16. In your opinion, what are the advantages and disadvantages of oritavancin compared to dalbavancin?

Advantages	Disadvantages

16.1. *If not addressed*, Are some of its disadvantages a barrier to the use of oritavancin?

☐ Yes ☐ No

16.1.1. If Yes, specify which one(s)

17. In your opinion, what will be the approximate number of patients for each product, one year after the commercialization of oritavancin according to the indications?

	Dalbavancin	Oritavancin	IV short half-life (vancomycin, etc.)	Oral short half-life	**Total**
Acute bacterial skin or soft tissue infections					∑1=
Bone and joint infections without implanted material					∑1=
Bone and joint infections with implanted material (prosthesis, plate, etc.)					∑1=
Osteomyelitis					∑1=
Infective endocarditis					∑1=
Prevention of postoperative infections					∑1=
Other (specify)					∑1=
Other (specify)					∑1=
	∑2=	∑2=			

17.1. For what reasons?

18. In your opinion, what would be the therapeutic prescription scheme for oritavancin in these different indications (treatment line, number of injections, dosage, frequency of injections, etc.)?

Acute bacterial skin or soft tissue infections	
Bone and joint infections without implanted material	
Bone and joint infections with implanted material (prosthesis, plate, etc.)	
Osteomyelitis	
Infective endocarditis	
Prevention of postoperative infections	
Other (specify)	
Other (specify)	

V. Economic considerations

19. Is special attention paid to the economic criteria of antibiotics in your establishment? (Multiple choices possible)

☐ Yes, at the pharmacy

☐ Yes, within the department

☐ No

Specify

20. Are long half-life antibiotic infusions ever performed outside of the hospital setting?

	Initiations	Renewals
Day Hospital	☐ Yes ☐ No	☐ Yes ☐ No
Home Hospitalization	☐ Yes ☐ No	☐ Yes ☐ No
Rehabilitation Care	☐ Yes ☐ No	☐ Yes ☐ No
Other (specify)	☐ Yes ☐ No	☐ Yes ☐ No

20.1. *If Yes*, How are these prescriptions financed?

Day Hospital	
Home Hospitalization	
Rehabilitation Care	
Other (specify)	

21. What is the most relevant price indicator for you for this type of medication?

☐ Price per injection

☐ Total cure price

22. In your opinion, could a price difference in favor of oritavancin compared to dalbavancin be beneficial to oritavancin? ☐ Yes ☐ No

If Yes,

22.1. What would be the market shares of each product depending on the price difference?

Price reductions	Dalbavancin	Oritavancin	Comments
Orita 5% cheaper than Dalba	%	%	
Orita 10% cheaper than Dalba	%	%	
Orita 15% cheaper than Dalba	%	%	
Orita 20% cheaper than Dalba	%	%	

23. In your opinion, if a generic were to come to market at about 70% cheaper than the original brand, what would be the approximate number of patients for each product? (Refer to the totals ∑1)

	Generic Dalbavancin	Dalbavancin	Oritavancin	IV short half-life antibiotics (vancomycin, etc.)	Oral short half-life antibiotics
Acute bacterial skin or soft tissue infections					
Bone and joint infections without implanted material					
Bone and joint infections with implanted material (prosthesis, plate, etc.)					
Osteomyelitis					
Infective endocarditis					
Prevention of postoperative infections					
Other (specify)					
Other (specify)					
